# The mental wellbeing of prison staff in England during the COVID-19 pandemic: A cross-sectional study

**DOI:** 10.3389/fpubh.2023.1049497

**Published:** 2023-03-03

**Authors:** Luke Johnson, Maciej Czachorowski, Kerry Gutridge, Nuala McGrath, Julie Parkes, Emma Plugge

**Affiliations:** ^1^School of Primary Care, Population Sciences and Medical Education, University of Southampton, Southampton, United Kingdom; ^2^Vulnerable People and Inclusion Health Directorate, UK Health Security Agency, London, United Kingdom; ^3^Division of Psychology and Mental Health, School of Health Sciences, Faculty of Biology, Medicine and Health, Centre for Women's Mental Health, Manchester Academic Health Science Centre, University of Manchester, Manchester, United Kingdom; ^4^Department of Social Statistics and Demography, Faculty of Social Sciences, University of Southampton, Southampton, United Kingdom

**Keywords:** COVID-19, prison, wellbeing, mental health, occupational health, health inequalities

## Abstract

**Background:**

COVID-19 is likely to have had an impact on the mental wellbeing of prison staff because of the high risk for infectious disease outbreaks in prisons and the pre-existing high burden of mental health issues among staff.

**Methods:**

A cross-sectional study of staff within 26 prisons in England was carried out between 20th July 2020 and 2nd October 2020. Mental wellbeing was measured using the Short-version of Warwick-Edinburgh Wellbeing Scale (SWEMWBS). Staff wellbeing was compared to that of the English population using indirectly standardised data from the Health Survey for England 2010–13 and a one-sample *t*-test. Multivariate linear regression modelling explored associations with mental wellbeing score.

**Results:**

Two thousand five hundred and thirty-four individuals were included (response rate 22.2%). The mean age was 44 years, 53% were female, and 93% were white. The sample mean SWEMWBS score was 23.84 and the standardised population mean score was 23.57. The difference in means was statistically significant (95% CI 0.09–0.46), but not of a clinically meaningful level. The multivariate linear regression model was adjusted for age category, sex, ethnicity, smoking status, occupation, and prison service region. Higher wellbeing was significantly associated with older age, male sex, Black/Black British ethnicity, never having smoked, working within the health staff team, and working in certain prison regions.

**Interpretation:**

Unexpectedly, prison staff wellbeing as measured by SWEMWBS was similar to that of the general population. Reasons for this are unclear but could include the reduction in violence within prisons since the start of the pandemic. Qualitative research across a diverse sample of prison settings would enrich understanding of staff wellbeing within the pandemic.

## Introduction

Worldwide, there are over 10 million people imprisoned (1). Early in the COVID-19 pandemic, prisons were classified as high-risk environments for outbreaks because of overcrowded conditions, frequent staff changes, and the regular movement of people in to, out of, and between prisons (2). Additionally, prison residents and staff have a high burden of chronic diseases and an over-representation of Black and ethnic minority groups, both of which are risk factors for severe COVID-19 infection (3, 4). Consequently, prisons in many countries implemented infection control measures which restricted residents' movement and access to visitors which likely to had a significant impact on mental wellbeing (2).

The prison system in England and Wales consists of 117 prisons, holding ~80,000 prison residents and employing ~53,000 staff (5). Prisons in England implemented infection control measures in March 2020 which led to the cessation of social visits and activities, face-to-face education, and training and employment opportunities (6). Restrictions on the numbers of people unlocked and numbers of people in exercise yards at any one time were also introduced to facilitate social distancing. However, these measures resulted in residents being confined to their cells for up to 23 h a day (2).

Prisons employ a diverse group of professionals, including prison officers, probation officers, administrators, nurses, doctors, psychologists, chaplains, and management. Most staff in English prisons are employed by Her Majesty's Prison and Probation Service (HMPPS), though nurses and doctors are employed by the National Health Service (NHS), and some employees are contract workers (such as maintenance workers). Prior to the pandemic, the available evidence suggested that prison staff in England had low wellbeing and a high burden of mental health issues (7–12). Factors associated with lower wellbeing included the demanding workload, high risk of violence and workplace injury, exposure to prison resident self-harm, and an oppressive work environment (13, 14). Most prison staff are classed as essential workers and have continued working in prisons throughout the pandemic (6). They have had to adapt to changes in prison regime, taking on new roles and responsibilities. By continuing to fulfil their duties at work, staff have put their own health, and that of their families, at risk (2). Recent findings suggested a higher burden of mental health symptoms in UK frontline workers than the general population during the first month of the March 2020 lockdown (15). Whilst it might be hypothesised that both the pandemic and changes to the prison regime have worsened wellbeing in prison staff in England, there is little evidence regarding this impact (2). The present study aims to examine the mental wellbeing of prison staff in England during the pandemic and determine factors associated with wellbeing. Such evidence is important for understanding how prison employers can ensure workforce resilience as we emerge from the pandemic and will provide useful learning for future pandemic preparation.

## Methods

This cross-sectional study uses data collected as part of the “COVID-19 in Prisons Study” (CiPS). CiPS aimed to examine the epidemiology of SARS-CoV-2 in prisons in England to inform policy and practise during the pandemic and recovery period. One objective was to examine the mental wellbeing of staff (16). CiPS was a repeated panel survey consisting of three rounds of data collection. Staff wellbeing was measured only in round two of data collection. Therefore, our study only uses cross-sectional data from round two of CiPS, collected between 1st September 2020 and 2nd October 2020. A purposive sample of 28 prisons in England was selected for their representativeness of closed prisons in function, security category, geographical area, staff population, resident population, and prior COVID-19 outbreak. All staff in the 28 selected prisons who were over 18 years old and regularly working on-site were eligible to participate in the study.

Staff wellbeing was measured using the Short version of the Warwick Edinburgh Mental Wellbeing Scale (SWEMWBS) (17, 18). SWEMWBS is a self-completed questionnaire consisting of seven positively-worded statements which assess the thoughts and feelings of participants over the last 2 weeks. Participants use a Likert scale to record their agreement with each statement. Examples of scale items are “I've been feeling optimistic about the future” and “I've been dealing with problems well”. The five-point Likert scale ranges from 1 = *None of the time* to 5 = *All of the time*. The scores are summed and transformed using a conversion table (19). The range of possible scores for SWEMWBS is 7–35 (higher scores imply higher wellbeing) and the minimum important level of difference in SWEMWBS score, defined as “of significance to the patient, member of the public, or the health professional”, has been estimated to be one point (18). The measure has good internal consistency, construct validity, and criterion validity and it has been validated in the UK population (17, 20). Using the sample SWEMWBS standard deviation (SD) (4.84), an α of 0.05, and a power of 90%, it was determined *post-hoc* using nQuery 8.7.0.0 that sample size was sufficiently large to detect a difference of one point in mean SWEMWBS scores between groups in all performed statistical tests (21).

A questionnaire was also administered to capture self-reported sociodemographics, height, weight, medical history, and symptoms (see Supplementary material for questionnaire). The comorbidities which were enquired about included heart disease, asthma, chronic obstructive pulmonary disease, chronic kidney disease and chronic liver disease. Additionally, participants were asked if they were on immune compromising medications and if a household member worked in a health or social care setting. The questionnaire collected the first four digits of a participant's home postcode, which was used to estimate the index of multiple deprivation (IMD) based on the IMD category most prevalent in that postcode. Each prison provided information on the prison environment and the number of prison staff and residents.

### Patient and public involvement

HMPPS staff and NHS England and NHS Improvement (NHSEI) were involved in the development and conduct of the study. People with experience of imprisonment were also represented on the study steering group.

### Statistical analyses

Stata version 16 (StataCorp LLC) was used for analysis (22). Descriptive statistics were generated to characterise the sample. For analysis, prisons were grouped into anonymised regions (R1–R13) to protect their identity. Characteristics (age and sex) of the participating sample were compared to the total staff population in prisons targeted for recruitment (the eligible population), as well as the total staff population in the English prison system. The sample mean SWEMWBS score was compared to the mean SWEMWBS score for the English population in the Health Survey for England (HSE) 2010–2013 (this was the most recent robust population data available), using summary data from a previous academic publication which was first age-sex standardised to the participating staff population distribution (17). Summary data was used as we did not have access to the raw HSE data on SWEMWBS.

Given the continuous nature of the outcome SWEMWBS score, univariate and multivariate linear regression models were used to identify associations with SWEMWBS score and quantify the differences in mean SWEMWBS scores between groups. Categorical variables were represented in models using dummy variables. Variables which were deemed clinically important a priori or showed statistical significance (*p* < 0.05) at the univariate stage were considered for inclusion in the final model. Age, sex, ethnicity, smoking status, and presence of comorbidities were judged clinically important because of known associations with wellbeing (17, 23). The multivariate linear regression model was created using backward selection, with all variables with one or more categories with a *p* < 0.05 being included in the final model. For regression modelling, age was dichotomised into 40 years and under and 41 years and over to divide the population approximately in half. Model assumptions for the multivariate linear regression model were checked, including visual inspection of the normality of the residuals and the use of Cook's distance to identify observations of high influence and leverage.

## Results

**Figure 1:**
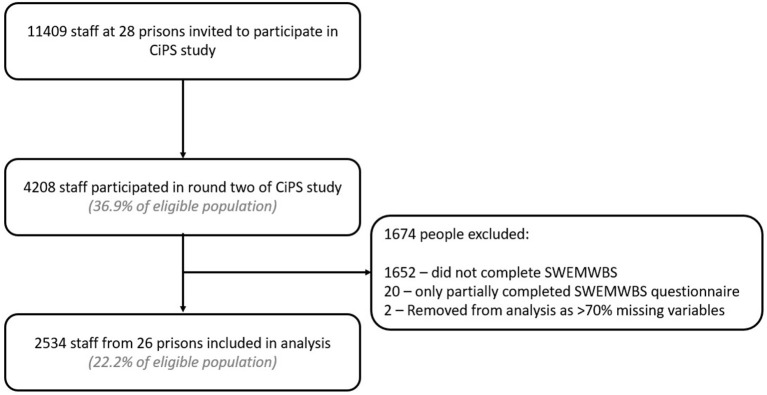
Survey flowchart: eligible population, recruitment, sample included in analysis.

Of 11,409 staff eligible to participate, there were 2,556 responses. Of these, 22 were removed because of missing answers to the SWEMWBS questionnaire, preventing calculation of the SWEMWBS score. A further two questionnaires which were completed but not linked to participant information were also excluded. Two thousand five hundred and thirty-four responses are therefore included in this analysis (22.2% of eligible staff). Nearly all eligible prisons (26 of 28) had staff who participated in the SWEMWBS questionnaire. Of these 26 prisons, the proportion of eligible staff participating varied between 0.2 and 52.1%. Of staff participating in round two, 60.2% completed the SWEMWBS questionnaire (Figure 1).

Characteristics (age and sex) of the included study sample were compared with the total staff population in the 28 prisons, and the total staff population in the English prison estate (Table 1) to assess for selection bias. Females were overrepresented in the sample compared to each population (*p* < 0.001 for both calculations), as were older staff members (*p* < 0.001 for both calculations).

**Table 1:** Comparison of age and sex distributions between sample, total staff in prisons targeted for recruitment, and total staff in English prison estate.

**Variable**	**Study sample (*n* = 2,534)**	**Total staff in prisons targeted for recruitment (*n* = 11,409)**	**χ^2^ *P*-value (sample vs. prisons targeted for recruitment)**	**Total staff in English prison estate (*n* = 53,473)**	**χ^2^ *P*-value (sample vs. prison estate)**
**Sex**			< 0.001^*^		< 0.001^*^
Male	1,187 (46.8%)	7,072 (62.0%)		27,049 (50.6%)	
Female	1,339 (52.8%)	4,337 (38.0%)		26,424 (49.4%)	
Missing	8 (0.3%)	0		0	
**Age categories**			< 0.001^*^		< 0.001^*^
16–24	135 (5.3%)	840 (7.4%)		3,456 (6.5%)	
25–34	567 (22.4%)	2,996 (26.3%)		13,565 (25.4%)	
35–44	550 (21.7%)	2,260 (19.8%)		11,926 (22.3%)	
45–54	725 (28.6%)	2,810 (24.6%)		13,036 (24.4%)	
55–64	497 (19.6%)	2,268 (19.9%)		10,427 (19.5%)	
65 and over	60 (2.4%)	235 (2.1%)		1,063 (2.0%)	

^*^Pearson's χ^2^ test.

The mean age was 43.7 years (range 19–76; SD 12.1) (Table 2). Most participants were of white ethnicity (93.3%) and the majority lived in areas in the three least deprived quintiles (83.0%). A substantial minority (29.0%) lived with someone who worked in a health or social care setting. Just under half were either smokers or ex-smokers (44.9%). The majority (66.7%) self-reported they were overweight, obese or extremely obese, and 19.3% had one or more comorbidities.

**Table 2:** Characteristics of sample and SWEMWBS score.

**Variable (*n* = 2,534)**	**Distribution *N* (%)**	**SWEMWBS score Mean (SD)**
**Age category (years)**		
≤30	464 (18.3%)	23.33 (4.61)
31–40	580 (22.9%)	23.50 (4.74)
41–50	613 (24.2%)	23.79 (4.89)
51–60	706 (27.8%)	24.31 (4.95)
≥61	174 (6.8%)	24.64 (4.90)
Missing	0	
**Sex**		
Male	1,187 (46.8%)	24.32 (5.14)
Female	1,339 (52.8%)	23.43 (4.53)
Missing	8 (0.3%)	
**Ethnicity**		
White	2,364 (93.3%)	23.78 (4.80)
Black/Black British	68 (2.7%)	25.27 (5.26)
Asian/Asian British	48 (1.9%)	24.66 (5.65)
Other ethnicity	53 (2.1%)	24.13 (4.80)
Missing	1 (0.0%)	
**BMI category**		
Underweight (< 18.5 kg/m^2^)	18 (0.7%)	24.54 (5.16)
Healthy (18.5–24.9 kg/m^2^)	658 (26.0%)	23.72 (4.49)
Overweight (25–29.9 kg/m^2^)	941 (37.1%)	23.74 (4.84)
Obese (30–39.9 kg/m^2^)	672 (26.5%)	24.17 (5.01)
Extremely obese (≥40 kg/m^2^)	78 (3.1%)	23.65 (4.87)
Missing	167 (6.6%)	
**Smoking status**		
Current smoker	344 (13.6%)	23.46 (5.08)
Ex-smoker	785 (31.0%)	23.64 (4.97)
Never smoked	1,396 (55.1%)	24.07 (4.70)
Missing	9 (0.4%)	
**Number of comorbidities**		
0	2,046 (80.7%)	23.90 (4.83)
1 or more	488 (19.3%)	23.61 (4.87)
**Immune compromising medications**		
Yes	113 (4.5%)	23.96 (5.01)
No	2,390 (94.3%)	23.84 (4.83)
Missing	31 (1.2%)	
**Occupation**		
Prison service staff	2,009 (79.3%)	23.75 (4.90)
Health staff	197 (7.8%)	24.51 (4.89)
Agency staff	259 (10.2%)	24.16 (4.69)
Probation service	66 (2.6%)	23.33 (2.99)
Missing	3 (0.1%)	
**IMD quintile of staff residence**		
1st and 2nd quintiles (most deprived)	425 (16.8%)	23.86 (4.88)
3rd quintile	1,086 (42.9%)	23.74 (4.77)
4th quintile	870 (34.3%)	24.07 (4.87)
5th quintile (least deprived)	148 (5.8%)	23.21 (5.11)
Missing	5 (0.2%)	
**Household member working in a health or social care setting**		
Yes	735 (29.0%)	23.79 (4.95)
No	1,791 (70.7%)	23.86 (4.79)
Missing	8 (0.3%)	
**HMPPS region** ^*^		
R1	176 (7.0%)	23.18 (4.93)
R2	292 (18.5%)	23.95 (4.51)
R3	594 (23.4%)	23.94 (5.06)
R4	486 (19.2%)	23.34 (4.10)
R5	69 (2.7%)	26.59 (5.67)
R6	89 (3.5%)	23.61 (4.48)
R7	67 (2.6%)	23.01 (4.54)
R8	1 (0.0%)	25.03 (n/a)
R9	52 (2.1%)	22.31 (4.25)
R10	99 (3.9%)	24.11 (4.71)
R11	176 (7.0%)	24.92 (5.46)
R12	175 (6.9%)	23.65 (4.76)
R13	258 (10.2%)	24.07 (5.24)
Missing	0	
**Prison security category**		
A (highest security)	709 (28.0%)	24.06 (5.17)
B	769 (30.4%)	23.83 (4.73)
C (lowest security)	778 (30.7%)	23.61 (4.56)
Female prisons	225 (8.9%)	24.34 (5.10)
YOIs	53 (2.1%)	22.36 (4.23)
Missing	0	
**Prison functional category**		
Trainer	1,624 (64.1%)	23.91 (4.92)
Local	858 (33.9%)	23.81 (4.70)
YOIs	53 (2.1%)	22.36 (4.23)
Missing	0	
**Prisoner/staff ratio**		
≤1	643 (25.4%)	23.51 (4.92)
1–2	1,016 (40.1%)	24.28 (4.95)
≥2	875 (34.5%)	23.58 (4.60)
Missing	0	

^*^Grouped into anonymised regions to protect identity.

BMI, body mass index; IMD, index of multiple deprivation; HMPPS, Her Majesty's Prison and Probation Service; SD, standard deviation; YOI, Young Offender Institutions.

The majority of staff directly worked for the prison service (79.3%) rather than a prison-related employer. Most participants worked in a male prison (89.0%); 8.9% worked in a female prison within the sample, and 2.1% worked in Young Offender Institutions (YOIs). Prisons were grouped by HMPPS region to take account of both geographical variation and variation in work culture—each region being managed by a Prison Group Director. Some regions only contained one prison in the sample, whilst others contained several. Unlike male prisons, female prisons and YOIs are not assigned security categories, and so are categorised separately within the prison security variable.

**Figure 2:**
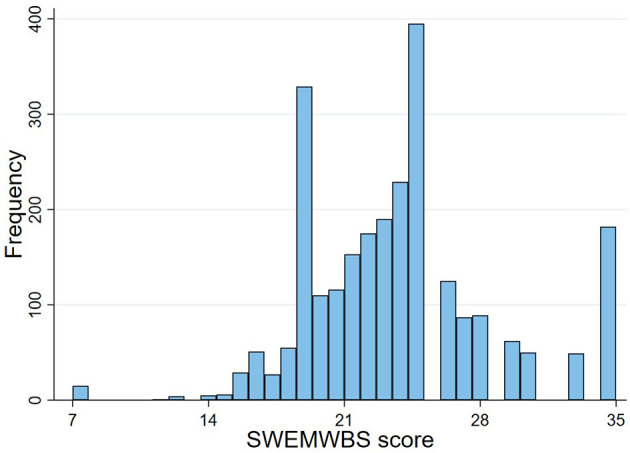
Histogram of SWEMWBS variable.

Descriptive statistics and mean SWEMWBS score in each category are presented in Table 2. The mean SWEMWBS score was 23.84 (range 7–35; SD 4.84). The distribution of scores is shown in Figure 2. The distribution was judged as approximately normal based on visual inspection and the similarity of the mean (23.84) and median (24.11). However, there were four total SWEMWBS scores of elevated frequency. It was ascertained that these totals related to people who had marked all SWEMWBS questions with the same answer, either 1, 2, 3, or 5. A score of ≤17 is highly correlated with mental illness (24)-−4.4% (*n* = 111) of participants scored ≤17.

The HSE standardised mean SWEMWBS score was 23.57. The prison staff sample had a statistically significantly higher mean SWEMWBS score (23.84) than the standardised HSE mean (*p* = 0.004; 95% CI 23.66, 24.03). However, this was less than the previously defined meaningfully important difference of one point.

In univariate linear regression analysis, SWEMWBS score was significantly associated with age, sex, ethnicity, smoking status, occupation, HMPPS region, prisoner functional category, and prisoner/staff ratio. The final multivariate linear regression model was adjusted for age category, sex, ethnicity, smoking status, presence of comorbidities, occupation, and HMPPS region (Table 3). Significantly higher SWEMWBS scores were associated with older age, male sex, Black/Black British ethnicity and staff occupation (specifically health staff compared to prison service staff). Additionally, HMPPS regions R5 and R11 were significantly associated with higher wellbeing as compared to R1 at the univariate and multivariate level (at multivariate level, β 3.81 (95% CI 2.47, 5.15, *p* < 0.001) and β 1.93 (95% CI 0.93, 2.94, *p* < 0.001), respectively). The only definitive meaningfully important difference in score (where confidence intervals did not cross minus one or one) came from working in the R5 as compared to R1 (95% CI 2.47, 5.15). The multivariate model did not explain the majority of variance (*R*^2^ = 0.0429).

**Table 3:** Primary model: Unadjusted and adjusted linear regression models for predicting SWEMWBS score.

	**Unadjusted models (*****N*** = **2,512)**			**Adjusted model**^**a**^ **(*****N*** = **2,512)**		

	β^*^	**95% CI**	***P*** **-value**	β^*^	**95% CI**	***P*** **-value**
**Age category**						
40 and under	0			0		
41 and over	0.70	(0.32, 1.09)	**< 0.001**	0.53	(0.14, 0.92)	**0.008**
**Sex**						
Female	0			0		
Male	0.90	(0.52, 1.28)	**< 0.001**	0.95	(0.53, 1.37)	**< 0.001**
**Ethnicity**						
White	0			0		
Black/Black British	1.62	(0.42, 2.83)	**0.008**	1.45	(0.25, 2.66)	**0.018**
Asian/Asian British	0.87	(−0.51, 2.26)	0.218	0.63	(−0.74, 2.00)	0.369
Other ethnicity	0.34	(−0.98, 1.66)	0.610	0.30	(−1.01, 1.60)	0.656
**Smoking status**						
Never smoked	0			0		
Current smoker	−0.62	(−1.19, −0.04)	**0.036**	−0.51	(−1.08, 0.07)	0.083
Ex-smoker	−0.44	(−0.87, −0.02)	**0.041**	−0.44	(−0.87, −0.02)	**0.040**
**No. of comorbidities**						
None	0			0		
1 or more	−0.31	(−0.79, 0.18)	0.213	−0.41	(−0.89, 0.07)	0.091
**Occupation**						
Prison service staff	0			0		
Health staff	0.77	(0.05, 1.48)	**0.035**	1.32	(0.60, 2.05)	**< 0.001**
Agency staff	0.42	(−0.21, 1.05)	0.192	0.62	(−0.01, 1.25)	0.053
Probation staff	−0.43	(−1.62, 0.76)	0.479	0.06	(−1.13, 1.24)	0.923
**HMPPS Region** ^**^						
R1	0			0		
R2	0.82	(−0.08, 1.73)	0.074	0.86	(−0.04, 1.76)	0.062
R3	0.80	(−0.01, 1.61)	0.054	0.87	(0.05, 1.69)	**0.038**
R4	0.19	(−0.64, 1.02)	0.656	0.31	(−0.52, 1.14)	0.465
R5	3.43	(2.09, 4.78)	**< 0.001**	3.81	(2.47, 5.15)	**< 0.001**
R6	0.46	(−0.77, 1.69)	0.465	0.67	(−0.55, 1.89)	0.280
R7	−0.15	(−1.50, 1.21)	0.832	0.06	(−1.30, 1.41)	0.932
R9	−0.76	(−2.27, 0.74)	0.319	−0.60	(−2.09, 0.89)	0.431
R10	0.96	(−0.23, 2.14)	0.115	0.91	(−0.27, 2.09)	0.130
R11	1.77	(0.76, 2.78)	**0.001**	1.93	(0.93, 2.94)	**< 0.001**
R12	0.52	(−0.49, 1.53)	0.316	0.57	(−0.43, 1.58)	0.263
R13	0.92	(−0.01, 1.85)	0.051	1.01	(0.08, 1.94)	**0.032**
**Prisoner functional category**						
Trainer	0			–		
Local	−0.11	(−0.51, 0.30)	0.606			
YOIs	−1.53	(−2.88, −0.18)	**0.018**			
**Prisoner/staff ratio**						
≤1	0			–		
1–2	0.79	(0.31, 1.26)	**0.001**			
>2	0.08	(−0.42, 0.57)	0.761			

Median and mean SWEMWBS scores for each independent variable category were very similar, suggesting they follow a normal distribution.

^a^Adjusted for age category, sex, ethnicity, smoking status, number of comorbidities, occupation, and HMPPS region.

^*^Beta coefficient for dependent outcome: WEMWBS score.

^**^Grouped into anonymised regions to protect identity.

CI, confidence interval; YOI, Young Offender Institution.

When checking model assumptions, the residuals were judged to be approximately normally distributed, with a slight positive skew (see Supplementary Figure S1). One hundred and fifty-nine observations were deemed influential using Cook's distance values (values > 4/n, where n is the number of observations (>0.00159) were considered influential). Of these, 114 (71.7%) were SWEMWBS scores of 7 or 35—people who had scored one or five for all questions. Another model (see Supplementary Table 1) was therefore created excluding observations with a SWEMWBS score of 7 or 35 as a sensitivity analysis. The same covariates were included to enable comparability with the primary model. Removing the influential observations improved the normality of the residuals (Supplementary Figure S2) but resulted in no important changes to the estimates in Table 3 or our conclusions.

## Discussion

This is one of the largest studies of prison staff health in England and it showed that the wellbeing of prison staff in England during the COVID-19 pandemic was similar to that of the general population pre-pandemic. Prison staff wellbeing was statistically significantly higher than that of the general population but this was not considered clinically meaningful, using the threshold of one-point on the SWEMWBS scale. It is important to note that we used summary data from the HSE 2010–13 (17) as a comparison as it was the most recent robust data available. It is likely that wellbeing among the general population has since changed because of the pandemic; SWEMWBS data from an online, quota-based questionnaire collected from March to May 2020—at the start of the first lockdown—suggested that general population wellbeing within the UK was lower than that at the time of the HSE 2010–13 (25). Therefore, the difference in wellbeing may be larger in comparison to the general population.

Within the prison staff population, higher wellbeing was associated with older age, male sex, Black/Black British ethnicity, never having smoked, working within the health staff team, and working in certain prison regions. Working in the R5 region represented the only meaningfully important improvement in wellbeing. Differences in wellbeing between HMPPS regions could be the consequence of geographical variation or, more likely, differences in aspects of workplace culture identified as important to prison staff wellbeing in previous systematic reviews (13, 14). Key aspects identified within these reviews include support from management, workload, and clarity of job role.

This study is the largest to date to explore the wellbeing of prison staff in the UK and one of only two examining their wellbeing during the COVID-19 pandemic (26). It uses SWEMWBS, a measure of wellbeing that has been validated in the UK population (17, 19). The sample was well-powered to detect meaningfully important differences in wellbeing. As far as the authors are aware, it is the first quantitative study in the UK to measure prison staff wellbeing across multiple prisons without using trade union membership as a sampling frame, which may bias the results as they represent only one sector of staff.

There are several limitations. Firstly, as a cross-sectional study, the findings represent association, not causation. With data collected at only one point, it is difficult to determine whether the COVID-19 pandemic truly affected staff wellbeing. Secondly, the low response rate of this study (22.2%) may have caused some selection bias; participants were more likely to be female and older than the population of all prison staff. Older prison staff were found to have higher wellbeing scores than younger prison staff, whereas female prison staff were found to have lower wellbeing scores than male staff. Two prisons did not provide any responses to the SWEMWBS questionnaire because of a miscommunication. Additionally, certain SWEMWBS scores were overly common within the expected distribution. These represented participants who had scored all questionnaire answers the same. This has not been reported in other studies using SWEMWBS, and it is uncertain why this happened here. However, a sensitivity analysis removing those who answered all questions as the lowest or highest value did not substantially alter results. We judged the appropriateness of our assumption of normality for our linear regression analyses by visual inspection of the residuals rather than results of the Shapiro-Wilk test because for large samples, such as ours, the statistical test can be extremely sensitive to small departures from normality (27). Finally, the study did not gather information on pre-existing mental illness, which might have helped improve the fit of the regression model, as mental health has been shown to be associated with SWEMWBS score (25).

This study found that prison staff wellbeing during the pandemic was similar to that of the general population prior to the pandemic. Only 4.4% of staff scores indicated possible mental illness. This contrasts with other cross-sectional studies examining staff wellbeing from prior to the pandemic which, using the General Health Questionnaire, estimated 56.6–95% of UK prison officers met criteria for potential mental illness (7–10). These studies all found prison staff wellbeing to be lower than that of the general population. Two of these studies were conducted in a single prison setting, whereas the other two used an online survey conducted through trade union channels. Findings also deviate from another study of UK prison officer wellbeing during the pandemic, in which 43% had symptoms of moderate or severe anxiety (26). However, the latter study suffered may be biassed because recruitment was conducted by email, through trade union channels, with a response rate of only 2.0%. Furthermore, with the exception of the GAD-7, none of the study measures were validated, and the study report was not peer-reviewed. Studies of other frontline workers during the pandemic have demonstrated mixed results. One study found that the wellbeing of UK social workers may have improved during the pandemic, potentially as a result of increased support and changes to working practises (28). Other studies found that the wellbeing of UK frontline workers may have deteriorated as the pandemic continued (15, 29), suggesting that our findings may not necessarily be representative of prison staff wellbeing for the entirety of the pandemic.

Reasons for the variations in results between this study and studies of prison staff wellbeing prior to the pandemic are unclear and could include the aforementioned differences in study methodology or a true difference in prison staff wellbeing. Such a difference in prison staff wellbeing could have been brought about through pandemic-related changes to prison job roles and operations. Prison residents spent large periods within their cells during the pandemic, which adversely impacted their mental health (2). However, it is also likely to have greatly impacted the role of prison staff (6). Moreover, the prison population in England and Wales has reduced by more than 5% since the start of the pandemic, with possible consequences on staff workload (5). Job demands and role clarity have been shown to be two key factors influencing staff wellbeing (8, 10, 30). Although the role of staff has likely been altered multiple times throughout the pandemic, the decrease in prison population and restriction in prison resident movement and activities may have led to a reduction in staff duties. This is likely to have contributed to the decline in assaults within prisons in England and Wales, which decreased by 40% in the 12 months following March 2020 (31). The number of serious assaults decreased by 47% and assaults on staff decreased by 24%. These reductions will likely have had a positive effect on staff wellbeing (30). Numbers of self-harm incidents recorded in England and Wales have reduced 22% in male prisons and 4% in female prisons within the same period. This change is partially the result of the reduction in prison populations—rates of self-harm incidents have only reduced 19% in male residents and, in fact, have increased 12% in female residents. Nevertheless, fewer self-harm incidents may also have benefited staff wellbeing (32–34).

Similar to the HSE 2010–13, within the multivariate model, SWEMWBS score was significantly higher for people of black ethnicity, older age, and non-smokers (17). Many of the factors previously found to be strongly associated with prison staff wellbeing were not measured directly within this study, perhaps explaining the model's lack of predictive ability. Such factors included job demands (8, 10), support from management (9), relationships with colleagues (8, 9), role clarity (8), detachment (35), and experiences of aggression (35). Some of these may be mediating the differences found between health staff and other prison staff, and between different prison regions. Risk factors for COVID-19 mortality were not associated with worse wellbeing. Index of multiple deprivation and BMI were not significantly associated with wellbeing, and older age and male sex were associated with increased wellbeing.

Although it is unclear why our study found prison staff wellbeing was similar to the pre-pandemic general population, existing literature provides an evidence basis for strategies to protect and improve staff wellbeing. Job role and demands should be considered, and whole-system efforts to address self-harm and assault should be reinvigorated to minimise the adverse impacts on both prison residents and staff. Focus should be placed on continuing to reduce the imprisoned population to ensure the safety of prison staff and residents and enabling the development of a genuinely rehabilitative culture. This is particularly important considering the lack of evidence that imprisonment reduces rates of recidivism (36), and that most people are imprisoned for non-violent offences, so are unlikely to pose a risk to the public (37).

Further research is needed to build on the findings of this study. Qualitative research across a broad range of prison staff groups and occupations would enrich what is currently known about prison staff wellbeing and the impact of the pandemic in prisons. Such research could look at the experiences of staff whilst working in the pandemic, and any changes in perception of their role or relationship to prison residents as a result. Additionally, more evidence is needed on interventions to improve prison staff wellbeing. In collaboration with key organisations such as HMPPS and the Prison Officers Associations, research across these areas would help to build more successful strategies for promoting staff wellbeing and lead to a culture of shared learning.

## Data availability statement

The datasets presented in this article are not readily available because all data is managed in accordance with the CiPS Privacy Notice and study protocol, as well as the General Data Protection Regulation (GDPR) guidelines. The safeguarding of participant confidentiality is of utmost importance and therefore any requests for data will be judged on a case-by-case basis by all relevant stakeholders. All shared data would be deidentified. For all requests regarding data, please contact the corresponding author. Requests to access the datasets should be directed to l.johnson@soton.ac.uk.

## Ethics statement

This study involving human participants was reviewed and approved by University of Southampton; Health Research Authority; Health and Care Research Wales. The patients/participants provided their written informed consent to participate in this study.

## Author contributions

JP, NM, MC, and EP designed the study. JP, NM, MC, EP, and KG were involved in study implementation. Analysis was undertaken by LJ, MC, and NM. LJ and EP drafted the manuscript, and all authors were involved in revising it. LJ affirms that the manuscript is an honest, accurate, and transparent account of the study being reported, that no important aspects of the study have been omitted, and that any discrepancies from the study as planned have been explained. All authors approved the final manuscript. All authors had full access to the data and final responsibility to submit for publication.
